# A novel recursive algorithm for the calculation of the detailed identity coefficients

**DOI:** 10.1186/s12711-015-0108-6

**Published:** 2015-04-29

**Authors:** Luis Alberto García-Cortés

**Affiliations:** Departamento de Mejora Genética, Instituto Nacional de Investigación Agraria, Ctra. de La Coruña km. 7.5, Madrid, 28040 Spain

## Abstract

**Background:**

A recursive algorithm to calculate the fifteen detailed coefficients of identity is introduced. Previous recursive procedures based on the generalized coefficients of kinship provided the detailed coefficients of identity under the assumption that the two individuals were not an ancestor of each other.

**Findings:**

By using gametic relationships to include three, four or two pairs of gametes, we can obtain these coefficients for any pair of individuals. We have developed a novel linear transformation that allows for the calculation of pairwise detailed identity coefficients for any pedigree given the gametic relationships. We illustrate the procedure using the well-known pedigree of Julio and Mencha, which contains 20 Jicaque Indians of Honduras, to calculate their detailed coefficients.

**Conclusions:**

The proposed algorithm can be used to calculate the detailed identity coefficients of two or more individuals with any pedigree relationship.

## Background

The 15 detailed states of identity were first described by Harris [1] and Gillois [2]. Throughout this paper, they will be referred to identity coefficients as described by Cockerham [3].

To circumvent the absence of recurrence rules to obtain identity coefficients, Karigl [4], following the rules for generalized kinship coefficients [1,3], obtained identity coefficients using a triangular linear transformation. This transformation provided the 9 condensed identity coefficients for any pair of individuals, but the 15 detailed identity coefficients can only be calculated using Karigl’s method under the assumption that neither of the two individuals is an ancestor of the other. To obtain generalized kinship coefficients, Lange and Sinsheimer [5] described an alternative way, which can calculate the detailed coefficients of identity without this limitation. Unfortunately, the implementation of the latter method is not a triangular linear transformation.

An alternative to using multiple kinship coefficients is the use of multiple gametic relationships. These relationships, called chromosome pedigrees by Donnelly [6], have been succesfully used to account for dominance in linear models [7-9]. Here, we use these multiple gametic relationships to develop a new method to calculate the 15 detailed identity coefficients.

## Generalized gametic relationships

The coancestry between two individuals *X* and *Y*, *ϕ*_*XY*_, is usually calculated following simple recurrence rules. These rules can be implemented using tabular methods or languages with recursive function support. To calculate the whole set of coancestries for a given pedigree, only two formulae are required [10]:

(1) $$\begin{array}{@{}rcl@{}} \phi_{XX} & = & \left(1+\phi_{FM}\right)/2, \\ \phi_{XY} & = & \left(\phi_{FY}+\phi_{MY}\right)/2, \end{array} $$

where *F* and *M* are the father and the mother of individual *X*, respectively. These equations operate successively over pairs (*X*,*Y*) where *X* is assumed to be more recent than *Y*. Let *x* and *x*^′^ be the paternally and maternally inherited copies at a given locus carried by individual *X* and *y* and *y*^′^ the corresponding copies carried by individual *Y*. The coancestry between *X* and *Y* can then be written as:

$${\fontsize{9.2}{12}\begin{aligned} \phi_{XY} = \frac{1}{4}\left[P\left(x\equiv y\right)+P\left(x\equiv y'\right)+P\left(x'\equiv y\right)+P\left(x'\equiv y'\right)\right], \end{aligned}} $$

where ≡ stands for identical by descent (IBD).

Analogous relationships have been described for gametes [6] as *ψ*_*ab*_=*P*(*a*≡*b*) and recurrence rules have been developed for their pairwise relationships [6,7]:

(2) $$\begin{array}{@{}rcl@{}} \psi_{aa} & = & 1, \\ \psi_{ab} & = & 1/2\left(\psi_{gb}+\psi_{hb}\right), \end{array} $$

where *a* and *b* denote two gametes in the pedigree. Both *g* and *h* are the direct ancestral gametes of *a*, that is, the gametes of the father or the mother if *a* is a paternal or maternal gamete, respectively. Although Equations (1) and (2) are closely related, they have different interpretations.

We use the *three-way* (*ψ*_*abc*_), the *four-way* (*ψ*_*abcd*_) and the *two-pair* (*ψ*_*ab*,*cd*_) gametic relationships as counterparts of the conventional generalized kinship coefficients [4]. These generalized gametic relationships correspond to the probability of three or four gametes to be IBD. Note that these multiple gametic relationships correspond to multiple gametic identities, regardless of the identity by descent with other gametes. For instance, for individuals *X* and *Y*, whose paternally and maternally inherited gametes are described above,

$$\begin{array}{@{}rcl@{}} {\fontsize{8.2}{12}\begin{aligned} \psi_{xx'y}=P\left(x \equiv x'\equiv y\right) = P\left(x \equiv x'\equiv y\equiv y'\right)+P\left(x \equiv x'\equiv y\not\equiv y'\right).  \end{aligned}} \end{array} $$

*ψ*_*ab*,*cd*_ is the probability that gametes *a* and *b* are IBD and simultaneously *c* and *d* are also IBD. For instance, for individuals *X* and *Y*,

$$\begin{array}{@{}rcl@{}} \psi_{xx',yy'} = P\left(x \equiv x'\equiv y\equiv y'\right)+P\left(x \equiv x'\not\equiv y\equiv y'\right).  \end{array} $$

The recursive formulae for the whole set of multiple gametic relationships are

(3) $$ {\small\begin{aligned} \psi_{aa} & = 1, \\[-2pt] \psi_{ab} & = \frac{1}{2}\left(\psi_{gb}+\psi_{hb}\right), \\[-2pt] \psi_{aaa} & = 1, \\[-2pt] \psi_{aab} & = \psi_{ab}, \\[-2pt] \psi_{abc} & = \frac{1}{2}\left(\psi_{gbc}+\psi_{hbc}\right), \\[-2pt] \psi_{aaaa} & = 1, \\[-2pt] \psi_{aaab} & = \psi_{ab}, \\[-2pt] \psi_{aabc} & = \psi_{abc}, \\[-2pt] \psi_{abcd} & = \frac{1}{2}\left(\psi_{gbcd}+\psi_{hbcd}\right), \\[-2pt] \psi_{aa,aa} & = 1, \\[-2pt] \psi_{aa,ab} & = \psi_{ab}, \\[-2pt] \psi_{ab,ac} & = \psi_{abc}, \\[-2pt] \psi_{aa,bc} & = \psi_{bc}, \\[-2pt] \psi_{ab,cd} & = \frac{1}{2}\left(\psi_{gb,cd}+\psi_{hb,cd}\right). \end{aligned}}  $$

For an easier implementation, Equation (3) can be summarized in a simple set of rules

1. In any *n-way* relationship, merge groups sharing a given gamete, that is, *ψ*_*ab*,*ac*_=*ψ*_*abac*_
2. In any *n-way* relationship, discard repeated gametes, i.e. *ψ*_*aabc*_=*ψ*_*abc*_ or *ψ*_*aa*,*bc*_=*ψ*_*a*,*bc*_.
3. Given that the probability of a gamete to be IBD to itself is 1, discard groups of identity including a single gamete, i.e. *ψ*_*a*,*bc*_=*ψ*_*bc*_.
4. Identities with a single gamete are 1 and identities with two or more founder gametes at the same group are 0.
5. Calculate $\psi _{a\theta }=\frac {1}{2}\left (\psi _{g\theta }+\psi _{h\theta }\right)$, where *θ* stands for any identity pattern and *a* for a gamete of the youngest individual. For instance $\psi _{ab,cd}=\frac {1}{2}\left (\psi _{gb,cd}+\psi _{hb,cd}\right)$ or $\psi _{abc}=\frac {1}{2}\left (\psi _{gbc}+\psi _{hbc}\right)$.

As long as these rules are correct and regardless of the number of gametes involved, they can be used to calculate identities involving more than two individuals.

## The detailed identity coefficients

The 15 detailed coefficients of identity for individuals *X* and *Y* can be calculated from the generalized gametic relationships by a simple linear transformation. We will consider the 15 partitions or identity states as described by Gillois [2] and Jacquard [11], i.e.,

$${\fontsize{8.8}{12}\begin{aligned} \begin{array}{llll} S_{1}:x\equiv x'\equiv y\equiv y' & S_{2}:x\equiv x'\equiv y\not\equiv y' & S_{3}:x\equiv x'\equiv y'\not\equiv y\\ S_{4}:x\equiv y\equiv y'\not\equiv x' & S_{5}:x'\equiv y\equiv y'\not\equiv x & S_{6}:x\equiv x'\not\equiv y\equiv y'\\ S_{7}:x\equiv x'\not\equiv y\not\equiv y' & S_{8}:x\not\equiv x'\not\equiv y\equiv y' & S_{9}:x\equiv y\not\equiv x'\equiv y'\\ S_{10}:x\equiv y\not\equiv x'\not\equiv y' & S_{11}:x\not\equiv y\not\equiv x'\equiv y' & S_{12}:x\equiv y'\not\equiv x'\equiv y\\ S_{13}:x\equiv y'\not\equiv x'\not\equiv y & S_{14}:x\not\equiv y'\not\equiv x'\equiv y & S_{15}:x\not\equiv y'\not\equiv x'\not\equiv y \end{array} \end{aligned}} $$

The detailed identity coefficients *δ*_*i*_ are the probabilities of each state *S*_*i*_ for a given pair of individuals.

Generalized kinships [4] are a linear transformation of the pairwise detailed identity coefficients. For instance, $\psi _{xx^{\prime }yy^{\prime }}$ requires the state *S*_1_ to be true, then we can write $\psi _{xx^{\prime }yy^{\prime }}=\delta _{1}$. Similarly, $\psi _{xx^{\prime }y}$ requires either *S*_1_ or *S*_2_ to be true and therefore $\psi _{xx^{\prime }y}=\delta _{1}+\delta _{2}$, and so on for all *ψ*. A set of 15 equalities is defined to set up the following linear system of equations.

(4) $$ {\small\begin{aligned} \left[\begin{array}{ccccccccccccccc} 1 & 0 & 0 & 0 & 0 & 0 & 0 & 0 & 0 & 0 & 0 & 0 & 0 & 0 & 0\\[-.5pt] 1 & 1 & 0 & 0 & 0 & 0 & 0 & 0 & 0 & 0 & 0 & 0 & 0 & 0 & 0\\[-.5pt] 1 & 0 & 1 & 0 & 0 & 0 & 0 & 0 & 0 & 0 & 0 & 0 & 0 & 0 & 0\\[-.5pt] 1 & 0 & 0 & 1 & 0 & 0 & 0 & 0 & 0 & 0 & 0 & 0 & 0 & 0 & 0\\[-.5pt] 1 & 0 & 0 & 0 & 1 & 0 & 0 & 0 & 0 & 0 & 0 & 0 & 0 & 0 & 0\\[-.5pt] 1 & 0 & 0 & 0 & 0 & 1 & 0 & 0 & 0 & 0 & 0 & 0 & 0 & 0 & 0\\[-.5pt] 1 & 1 & 1 & 0 & 0 & 1 & 1 & 0 & 0 & 0 & 0 & 0 & 0 & 0 & 0\\[-.5pt] 1 & 0 & 0 & 1 & 1 & 1 & 0 & 1 & 0 & 0 & 0 & 0 & 0 & 0 & 0\\[-.5pt] 1 & 0 & 0 & 0 & 0 & 0 & 0 & 0 & 1 & 0 & 0 & 0 & 0 & 0 & 0\\[-.5pt] 1 & 1 & 0 & 1 & 0 & 0 & 0 & 0 & 1 & 1 & 0 & 0 & 0 & 0 & 0\\[-.5pt] 1 & 0 & 1 & 0 & 1 & 0 & 0 & 0 & 1 & 0 & 1 & 0 & 0 & 0 & 0\\[-.5pt] 1 & 0 & 0 & 0 & 0 & 0 & 0 & 0 & 0 & 0 & 0 & 1 & 0 & 0 & 0\\[-.5pt] 1 & 0 & 1 & 1 & 0 & 0 & 0 & 0 & 0 & 0 & 0 & 1 & 1 & 0 & 0\\[-.5pt] 1 & 1 & 0 & 0 & 1 & 0 & 0 & 0 & 0 & 0 & 0 & 1 & 0 & 1 & 0\\[-.5pt] 1 & 1 & 1 & 1 & 1 & 1 & 1 & 1 & 1 & 1 & 1 & 1 & 1 & 1 & 1 \end{array}\right]\left[\begin{array}{c} \delta_{1}\\[-.5pt] \delta_{2}\\[-.5pt] \delta_{3}\\[-.5pt] \delta_{4}\\[-.5pt] \delta_{5}\\[-.5pt] \delta_{6}\\[-.5pt] \delta_{7}\\[-.5pt] \delta_{8}\\[-.5pt] \delta_{9}\\[-.5pt] \delta_{10}\\[-.5pt] \delta_{11}\\[-.5pt] \delta_{12}\\[-.5pt] \delta_{13}\\[-.5pt] \delta_{14}\\[-.5pt] \delta_{15} \end{array}\right]=\left[\begin{array}{c} \psi_{xx'yy'}\\[-.5pt] \psi_{xx'y}\\[-.5pt] \psi_{xx'y'}\\[-.5pt] \psi_{xyy'}\\[-.5pt] \psi_{x'yy'}\\[-.5pt] \psi_{xx',yy'}\\[-.5pt] \psi_{xx'}\\[-.5pt] \psi_{yy'}\\[-.5pt] \psi_{xy,x'y'}\\[-.5pt] \psi_{xy}\\[-.5pt] \psi_{x'y'}\\[-.5pt] \psi_{xy',x'y}\\[-.5pt] \psi_{xy'}\\[-.5pt] \psi_{x'y}\\[-.5pt] 1 \end{array}\right] \end{aligned}}  $$

The coefficient matrix in Equation (4) is triangular and is equivalent to formula (8) in [4]. Nevertheless, the right hand side here includes the generalized gametic relationships instead of their kinship counterparts.

## An example: the detailed identity coefficients of the Jicaque Indians

**Figure 1:**
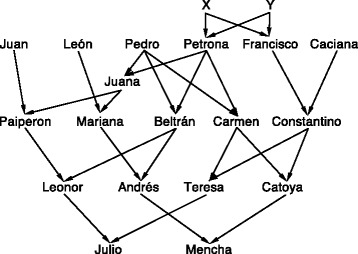
The pedigree of the Jicaque Indians Julio and Mencha.

We reanalyzed the pedigree of the Jicaque indians Julio and Mencha [12] presented in Figure 1 and previously analyzed in [4,11,13]. Previous analyses have focused on the identity coefficients between Julio and Mencha and between two of their progeny. Table 1 shows these results and those of two other pairs: Julio vs. one of their progeny and Mencha vs. one of their progeny.

**Table 1:** **Detailed coefficients of identity for four pairs involving Julio, Mencha and their progeny**

	**Julio-Mencha**	**Progeny-progeny**	**Julio-progeny**	**Mencha-progeny**
*δ*_1_	0.01025	0.06580	0.03467	0.03467
*δ*_2_	0.02393	0.04333	0.08252	0.00000
*δ*_3_	0.02490	0.04333	0.00000	0.08252
*δ*_4_	0.02393	0.04333	0.06665	0.06665
*δ*_5_	0.02490	0.04333	0.08228	0.08228
*δ*_6_	0.00708	0.00729	0.00000	0.00000
*δ*_7_	0.05103	0.02383	0.00000	0.00000
*δ*_8_	0.05103	0.02383	0.00000	0.00000
*δ*_9_	0.05127	0.24713	0.08228	0.06665
*δ*_10_	0.10937	0.15900	0.29248	0.00000
*δ*_11_	0.16992	0.15900	0.00000	0.29248
*δ*_12_	0.00708	0.00729	0.06665	0.08228
*δ*_13_	0.05103	0.02383	0.00000	0.29248
*δ*_14_	0.05103	0.02383	0.29248	0.00000
*δ*_15_	0.34326	0.08582	0.00000	0.00000

Program source codes written in Fortran 90 and examples are available at https://github.com/agarcor21/IdentityCoefficients.

## Discussion

The identity by descent of two gametes [6-9] is conceptually simpler than the kinship between individuals because it avoids any random sampling of genes. Although the number of gametes is larger than the number of individuals, its simple definition results in more intuitive recursive formulae and easier calculations.

Here, we have provided a novel method to derive the detailed identity coefficients [1,2]. Karigl derived a simple transformation to calculate these coefficients from the multiple kinship coefficients, which provided the 15 identity coefficients when the individuals were not ancestor-descendant related. Lange and Sinsheimer [5] first provided exact equations for these 15 coefficients without any assumption. Depending on the purpose of the study, researchers have used 7 or 9 condensed identity coefficients. For instance, in order to apply the identity coefficients to dominance models, 7 condensed coefficients can be used [8,9]. To our knowledge, the algorithm proposed in [9] has never been used to calculate the 15 detailed coefficients, but the formulas presented in their paper could be used to obtain them in a direct way.

We have proposed using multiple gametic relationships to calculate the 15 detailed identity coefficients. The linear transformation in this paper is similar to equation 8 of Karigl’s approach [4]. Reordering the rows in Equation (4) to follow Karigl’s pattern [4], both formulae only differ on the right hand side. Note that the meaning of both formulae is rather different.

The procedure presented here has been successfully implemented on small pedigrees. In large data sets, the number of calls to the recursive function will depend on the number of generations and the structure of the pedigree. Implementing the procedure in such scenarios is beyond our goal, but path counting or graph theory-based methods [14,15] have been developed to improve the computing efficiency of this calculation.
